# Modeling emergency department visit patterns for infectious disease complaints: results and application to disease surveillance

**DOI:** 10.1186/1472-6947-5-4

**Published:** 2005-03-02

**Authors:** Judith C Brillman, Tom Burr, David Forslund, Edward Joyce, Rick Picard, Edith Umland

**Affiliations:** 1 Department of Emergency Medicine, MSC10 5560, 1 University of New Mexico, Albuquerque NM 87131-0001, USA; 2 Mail Stop F600, Los Alamos National Labs, Los Alamos, New Mexico 87545, USA; 3 Mail Stop T006, Los Alamos National Labs, Los Alamos, New Mexico 87545, USA; 4 Mail Stop F607, Los Alamos National Labs, Los Alamos, New Mexico 87545, USA

## Abstract

**Background:**

Concern over bio-terrorism has led to recognition that traditional public health surveillance for specific conditions is unlikely to provide timely indication of some disease outbreaks, either naturally occurring or induced by a bioweapon. In non-traditional surveillance, the use of health care resources are monitored in "near real" time for the first signs of an outbreak, such as increases in emergency department (ED) visits for respiratory, gastrointestinal or neurological chief complaints (CC).

**Methods:**

We collected ED CCs from 2/1/94 – 5/31/02 as a training set. A first-order model was developed for each of seven CC categories by accounting for long-term, day-of-week, and seasonal effects. We assessed predictive performance on subsequent data from 6/1/02 – 5/31/03, compared CC counts to predictions and confidence limits, and identified anomalies (simulated and real).

**Results:**

Each CC category exhibited significant day-of-week differences. For most categories, counts peaked on Monday. There were seasonal cycles in both respiratory and undifferentiated infection complaints and the season-to-season variability in peak date was summarized using a hierarchical model. For example, the average peak date for respiratory complaints was January 22, with a season-to-season standard deviation of 12 days. This season-to-season variation makes it challenging to predict respiratory CCs so we focused our effort and discussion on prediction performance for this difficult category. Total ED visits increased over the study period by 4%, but respiratory complaints decreased by roughly 20%, illustrating that long-term averages in the data set need not reflect future behavior in data subsets.

**Conclusion:**

We found that ED CCs provided timely indicators for outbreaks. Our approach led to successful identification of a respiratory outbreak one-to-two weeks in advance of reports from the state-wide sentinel flu surveillance and of a reported increase in positive laboratory test results.

## Background

Traditional public health surveillance for specific conditions is unlikely to quickly identify a disease outbreak. Emergency department (ED) data appears to have the potential for more timely disease surveillance [1-6]. In non-traditional surveillance, signs of an outbreak might include an increase in ED visits for respiratory, gastrointestinal or neurologic chief complaints (CC).

Crucial to any surveillance is an understanding of normal patterns in the data. Utilization patterns in the ED are thought to be difficult to predict, due to large variability that arises in part because EDs are required to medically evaluate and stabilize everyone who requests care. Therefore, ED visit rates cannot be controlled by insurers, institutions or policies.

Many "drop in" surveillance systems have been hampered by a lack of knowledge of baseline activity [7]. Other systems have used short-term moving averages of the recent past to predict current usage [8], but this does not provide optimal performance when day-of-the-week or seasonal effects are smoothed away by the averaging.

Some "active" ED surveillance systems provide on-going data collection. The EMERGEncy ID NET [9] and RSVP [10] function as sentinel surveillance systems where data from a small sample used may not represent the overall occurrence of disease. If data collection is inconsistent, it does not provide reliable information about syndrome incidence.

This paper reviews our experience with an operational near-real-time surveillance system, the Bio-Surveillance Analysis, Feedback, Evaluation and Response (B-SAFER) system. B-SAFER is the result of collaboration between the Los Alamos National Laboratory, the University of New Mexico Health Sciences Center, and the New Mexico Department of Health. Medical surveillance systems such as B-SAFER require considerable expertise in computer science and systems integration for their design and architecture, to comply with security and privacy issues, and to ensure timely flow of information [11,12]. These systems also require medical and epidemiological expertise to identify appropriate items to monitor for anomalous events and to understand their significance.

## Methods

### Setting

This observational study uses data from the Emergency Center of the University Hospital, Albuquerque NM (UH), a tertiary-care county-university health sciences center. The Emergency Center includes pediatric and adult emergency departments, an urgent care center, a trauma center and an observation unit. There are roughly 200 patient visits per day, or 73,000 per year, representing 36 % of Emergency Department visits in Albuquerque. This study was approved by the Institutional Review Boards of the University of New Mexico Health Sciences Center and Los Alamos National Laboratory.

### Data stream

The data is from the computerized ED patient tracking system in place since 1994. Data in the system includes: date and time of arrival and discharge, age, sex, chief complaints, discharge diagnoses and disposition. CCs are recorded by the nurse at the time of triage and entered into the system by a clerk. The clerk may select from a drop-down menu of complaints or may enter the complaints as free text. The menu option is rarely used because clerks find free-text entry more flexible and convenient.

We group daily CC counts into seven categories: respiratory, gastrointestinal (GI), undifferentiated infection (UDI), lymphatic, skin, neurological, and "other" (Table 1). The "other" category includes all visits except those in the first six categories. These grouping categories are also used by other surveillance programs, such as ESSENCE (Walter Reed Army Institute of Research) and the Real-time Outbreak Detection System ((RODS, University of Pittsburgh) [11,13,14]. Our grouping scheme is provided in Table 1.

**Table 1:** B-SAFER dictionary for matching chief complaints to body systems

**Respiratory**	**Gastro-intestinal**	**Neurologic**	**Skin**	**Lympatic**	**UDI (undifferentiated infection) **
Breath Bronchiolitis Chest congestion Chest pain Cold Congested Congestion Cough Croup Flu Headache Laryngitis Pneumonia Respiratory Sinus Stuffy nose Throat	Abdominal Pain Abdomen/back pain Abdomen pain Abdominal cramps Abdominal pain Blood in stool Diarrhoea Food poisoning Hepatitis Jaundice Stomach pain Vomit Nausea Non responsive	Altered mental status Anxious Confusion Difficulty Talking Difficulty thinking Difficulty Walking Disoriented Drowsy Facial droop Facial weakness Hyper Loss of consciousness Mental Nervous Numbness Paralysis Seizure Slurred speech Sores Stroke Swallowing Syncope Thinking slow Tingling Trouble talking Trouble thinking Trouble walking Unresponsive Weak	Abscess Abnormal Skin Blisters Bug Bites Cellulitis Chicken pox Dermatitis Insect bite Itching Pox Rash Skin redness Skin swelling Tick bite	Arm pit Glands Lumps Lumps in neck Neck Nodes Red streaks Skin streaks Weak	Achy Body aches Body sores Fatigue Fever Fussy Infection Tired

To obtain this scheme, we reviewed a frequency table of all CCs which occurred at least 5 times over nine years and assigned relevant CCs to groups, as was done for example in [15] and [16]. Key words were selected to capture multiple chief complaints containing that word. For instance "breath" captures "shortness of breath", "trouble breathing", "I can't breathe", "can't catch breath", "breathing problems", etc. Common abbreviations were also included as key words. The groupings were then reviewed by the project medical advisory board which included epidemiologists, infectious disease, emergency and occupational health physicians.

Each CC is assigned to a group when the first match was made between a word in the CC and a word in our CC dictionary. There were no examples of negative chief complaints such as "no cough", in our nine-year database, so we did not develop a system for handling these. Any negative complaint would have been classified into the category of the key word.

### First order model

The training set for model development was retrospective cohort data from 2/1/94 through 5/31/02. We used least squares regression in started log scale to fit trends, seasonal effects, and day-of-week effects. The "started log" is the logarithm of one plus the number of daily CCs. We add one before taking the logarithm to avoid problems with taking the logarithm of zero counts. The started log scale results in more symmetrically distributed forecast errors with variance that is much less dependent on the mean count. Results are back-transformed to natural scale for display. Error bars behave as desired (widening when the CC count increases) and skewing is commensurate with the magnitudes of the baseline values.

A model that incorporates the above effects is

*S(d) *= [Σ_*i *_*c*_*i *_× *I*_*i*_*(d)*] + [*c*_8 _+ *c*_9 _× *d*] + [*c*_10 _× *cos*(2π*d */ 365.25) + *c*_11 _× *sin*(2π*d */ 365.25)]

where

a) *S*(*d*) denotes the started log of the number of chief complaints) for day *d*, where "Day 1" is taken as February 1, 1994,

b) [Σ_*i *_*c*_*i *_× *I*_*i*_*(d)*] captures the day-of-the-week effect, where the sum is over the indices *i *= 1 to 7 and *I*_*i*_*(d) *denotes the indicator function for day *d*, i.e., *I*_*i*_*(d) *equals 1 when day *d *is the *i-*th day of the week and equals zero otherwise, and the seven model coefficients {*c*_*i*_} are constrained to sum to zero,

c) [*c*_8 _+ *c*_9 _× *d*] captures a long term linear effect,

d) [*c*_10 _× *cos*(2π*d */ 365.25) + *c*_11 _× *sin*(2π*d */ 365.25)] captures the seasonal component, where the average number of days per year is 365.25, with the model coefficients *c*_10 _and *c*_11 _dictating the time and amplitude of the seasonal effect.

**Figure 1:**
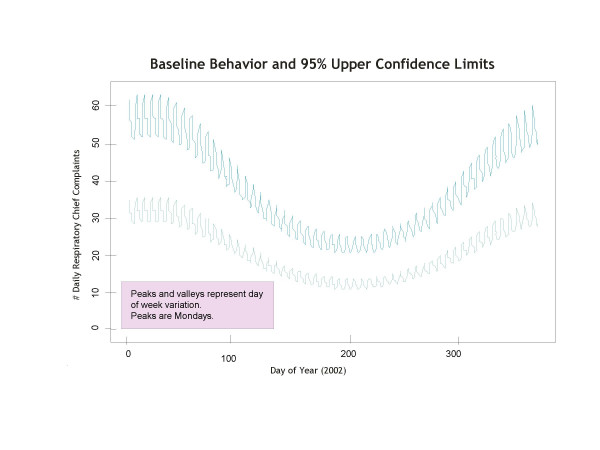
**Predicted Respiratory Complaints. **The predicted respiratory complaints and corresponding upper confidence limits illustrates seasonality and day-of-week effects.

This model arises from transforming the counts to started log scale and adding day-of-the-week effects to the regressor variables in a cyclical regression model [16,17]. Application of the model allows for coefficients to be set to zero when the corresponding constituent effects are not statistically apparent. For example, the coefficient *c*_9 _is zero for complaint categories that do not exhibit linear long term trends, and the coefficients *c*_10 _and *c*_11 _are zero for categories which do not exhibit seasonality. A plot of the predicted respiratory complaints and corresponding upper confidence limits from this model is given in Figure 1, illustrating how the constituent effects interact to yield baseline predictions. The predictions were obtained using ordinary least squares for parameter estimation and then back transforming from started logs to natural scale.

### Evaluation of goodness of fit

As with all statistical models, it is important to assess goodness of fit. A careful residual analysis reveals trends in the forecast errors, the most important of which follow from the one-size-fits-all character of the model. That is, the first order model postulates that complaint activity peaks with the same magnitude and at exactly the same time of year from season to season. Such postulated behavior is only approximately true, and the season-to-season differences lead to the trends in residuals from the model.

For example, if a peak of respiratory complaints occurs later than average in the year, then the baseline will initially over-predict (in anticipation of an average peak time) and then under-predict (when the season's peak actually occurs). Similarly, if the amplitude of a season's peak is higher or lower than the historical average amplitude, predictions will be consistently too low or too high near the peak. We return to this subject in the section on hierarchical modeling.

### Near real time monitoring: Page's test

By comparing CC counts to predictions and confidence limits, anomalies can be identified [18,19]. Extra counts could arrive all on one day, or appear as a gradual increase starting at some particular time, or arrive sporadically, persist at a constant level for a fixed duration, etc. The best statistical test for detecting extra counts depends on the pattern of extra counts, so there cannot be a single best test for detecting all possible anomalous patterns.

A particular test, based on Page's statistic, is optimal for detecting a constant excess above baseline when the start time and duration of the excess is unknown [20]. This test also has competitive power compared to other sequential tests to detect other anomalous patterns. For these reasons, Page's statistic is widely used in statistical process control and has been proposed in the context of disease surveillance [21]. We recommend Page's statistic unless specific anomalous patterns are suspected, in which case a specialized test could be developed.

Page's statistic is a type of cumulative sum, which we denote as *P*(*d)*. On each day *d*, the forecast error ε_*d *_between the started log of the observed number of complaints and the started log of the baseline prediction is computed for each complaint category. The standard deviation *s*_*d *_of ε_*d *_is computed as well. Then Page's statistic is calculated for day *d *as

*P*(*d*) = maximum of 0 and [*P*(*d*-1) + ε_*d*_/*s*_*d *_- 1/2].

If *P*(*d*) becomes too large, then the observed complaint levels are significantly greater than the baseline predictions and complaint levels are deemed anomalously high. Here, the phrase "too large" is formally defined in terms of the desired false positive rate for monitoring, and the threshold value for *P*(*d*) is calibrated using empirical data to account for model prediction errors.

### Comparison to other data streams

A comprehensive comparison to other data streams is beyond our scope. Briefly, we compared our respiratory CC counts to existing influenza data for the 2002–2003 influenza season. We evaluated New Mexico (NM) Department of Health sentinel influenza surveillance data reported weekly by approximately 20 clinics. When the week-long monitoring period is combined with the time needed for compilation and dissemination, notification of an outbreak early in one week is often not formally received until two weeks after it occurred. We obtained virology laboratory data from routine clinical and surveillance testing of respiratory specimens reported by three laboratories that conduct at least 75% of the clinical virology testing for NM. Such data streams have their own timeliness issues, due in part to culturing of samples.

## Results

ED data from 2/1/94 – 5/31/02 is used as training data for least squares fitting to establish control limits; data from 6/1/02 – 53/31/03 is then used in near real time surveillance. Approximately, 17% of the complaints fall into the respiratory category, 10% gastrointestinal, 6% undifferentiated infection, 3% skin, 3% neurological, 1% lymphatic and 60% "other."

### Day-of-week effects

**Figure 2:**
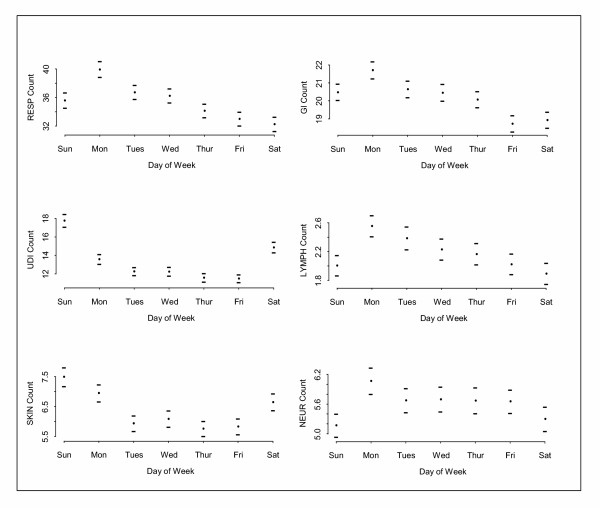
**Day-of-week effects for each CC category. **The average day-of-week effect with corresponding error bars for six CC categories.

For all systemic complaint categories there are day-of-week differences. See Figure 2. For five of the seven categories, there are more visits on Monday than on any other day; UDI and skin peak on Sunday. Weekly minimums occur later in the week: on Thursday for skin, Friday for GI and UDI, and Saturday for respiratory, neurologic and lymphatic. In some cases, there is a high-to-low trend as the week progresses.

For respiratory and UDI complaints, there is an average difference of 7 cases per week between Monday and Friday, and the weekly differences conform to a bell-shaped statistical distribution. While day-of-week effects are statistically significant in all categories owing to the size of the data set, in some categories there are so few complaints that the difference is of no practical consequence. For example, in the lymphatic and neurologic categories, the average difference between the weekly peak (Monday) and weekly minimum (Saturday) is less than one case per day. Certain other daily effects may exist, e.g. holiday effects [22], but sample sizes for UH data are not large enough to detect them.

### Seasonal effects

**Figure 3:**
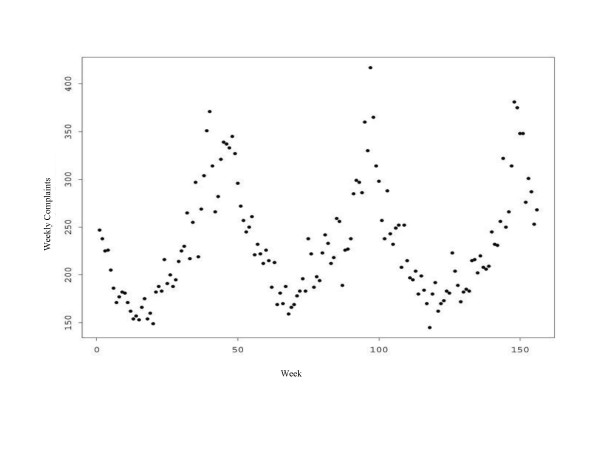
**Annual cycles in Respiratory Complaints. **Annual cycles in respiratory complaints (by week) for the past three flu seasons, from 2000–1 through 2002–3.

As is well known, there are annual cycles of respiratory complaints with peaks in January or February. Figure 3 demonstrates these cycles in our data for respiratory complaints; the cycles are similar but less pronounced for UDI complaints.

### Long term trends

**Figure 4:**
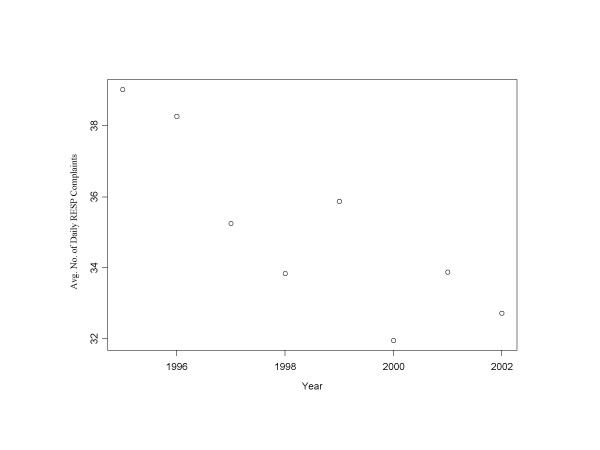
**Average daily number of respiratory complaints by calendar year. **The average daily number of respiratory CCs decreases over the training data.

Total ED visits increased over the study period by 4%, in part reflecting the population increase of about 1.5 % per year for the metropolitan area. Rates for most of the complaint categories have changed over the eight years that data has been collected. Respiratory complaints show a decrease of roughly 20% over the monitoring period (Figure 4), illustrating that long term averages need not reflect current or future behavior. Skin-related complaints also show a slight decrease, while increases are observed in nearly all other categories. Only lymphatic complaints do not appear to change over the monitoring period. Had there been a complaint category where a nonlinear trend were clearly present, either the *c*_9 _component would have been modified periodically, or a nonlinear model would have been used.

### Surveillance

#### Patterns observed in the Test Year

**Figure 5:**
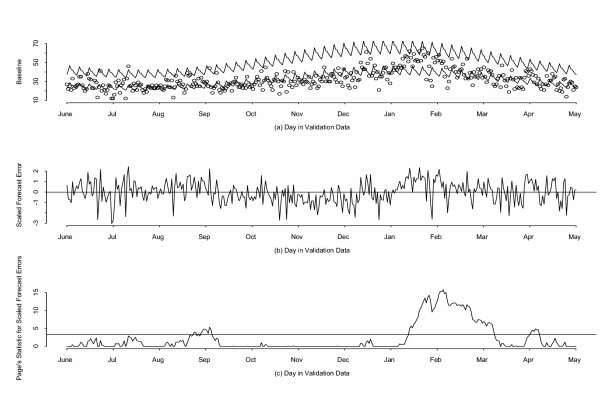
**Results on validation data for Respiratory Complaints. **Prospective data (June 1, 2002 through May 31, 2003 (a) Daily, predicted, and upper control limit for respiratory counts ; (b) Scaled forecast errors for respiratory counts; (c) Page's statistic applied to the same forecast errors. A control value of 3.3 bfor Page's statistic results in an approximate theoretical 2.5% false alarm rate when forecast errors are Gaussian.

We focus on respiratory CCs because they have the strongest season-to-season variation, which makes them the most challenging to predict, and because respiratory is thought to be one of the most important bioterrorist categories. Overlaying the data streams on the baseline plot in real time allows for visual inspection of the results, similar to that for a standard control chart. Figure 5a shows the daily counts, the baseline prediction, and the upper control limit (for a 2.5% false alarm rate) for the respiratory category. Figure 5b shows the scaled forecast errors (in the started log scale) for the prospective data. Both figures reveal a later-than-average flu season, as does Figure 5c, showing large values of Page's statistic *P(d)*. These plots illustrate how a misfit to the one-size-fits-all model can produce systematic trends in surveillance data. This subject is revisited in the section on hierarchical modeling.

The peak in respiratory CCs and the elevated *P(d) *preceded a rise in reports from the state-wide influenza sentinel surveillance system. A similar pattern, delayed by several weeks, was found in the rise of requests for laboratory tests for influenza. ED CCs also preceded New Mexico reporting of deaths from pneumonia and influenza. We conclude that surveillance using the first order model is sufficiently sensitive to mild departures from baseline activity and that it can provide timely notification relative to traditional surveillance.

#### Simulated outbreaks

In a simulation study we injected *K *extra respiratory CC counts beginning at random days during the test year, with the simulated outbreak lasting from 1 to 10 days, from 2 to 10 days, and exactly 1 day. Generally, departures of approximately 3 or more standard deviations from the baseline model should be detected with high probability. The simulated per-day shift above the baseline prediction ranged from 1 to 5 standard deviations in our simulations, so testing one day at a time could fail to detect those outbreak having small per-day shifts. Also, because of the pattern in the residuals near each seasonal peak, we considered EWMA (exponentially weighted moving average, see the Discussion) as one way to modify the current forecast on the basis of errors in the recent past.

In Table 2 we give the fraction of simulations (out of 1000) in which the Page statistic exceeded its threshold of 3.3 for the null model, baseline model, and for the same models modified by the EWMA procedure. For comparison to one-day-at-a-time testing, we also give the fraction of simulations in which the maximum forecast error that occurred during the outbreak exceeded its 2.5% false alarm rate threshold of 1.96. We see that Page's test outperforms the one-at-a-time test and that the EWMA modification does not improve anomaly detection because of its tendency to underestimate the size of multiple-day outbreaks. However, if we restrict attention to those outbreaks that last only one day, then one-at-a-time testing is better (for each of the models), as we would expect. Compare the baseline model results to the null model (which uses the average CC count in the training data to predict the test data) results to gauge the benefit of fitting the baseline model. Of course the null model is not acceptable regardless of its performance in this context because it ignores the trend (which causes the null model to be biased high for the respiratory CCs), day-of-week effects, and seasonality.

**Table 2:** The fraction of simulations (out of 1000, so the 95% confidence limit is approximately ± 0.03) in which the Page statistic (or the one-at-a-time statistic) exceeded its 2.5% false alarm threshold during the simulated outbreak for the baseline model, the baseline model with residuals modified by EWMA, the null model, and the null model with residuals modified by EWMA.

Outbreak duration	Test	Baseline	Baseline + EWMA	Null	Null + EWMA
1–10 days	Page	0.46	0.21	0.28	0.17
1–10 days	One-at-a-time	0.37	0.31	0.17	0.31
>1 day	Page	0.42	0.20	0.30	0.14
>1 day	One-at-a-time	0.31	0.25	0.13	0.26
1 day	Page	0.44	0.36	0.30	0.45
1 day	One-at-a-time	0.71	0.70	0.47	0.68

## Discussion

### Models

Long-term trends can occur in surveillance data for multiple reasons. Changes may occur in: local resources (more or specialty EDs), access and reimbursement practices (facilities change which insurance plans with which they are associated, major shifts in insurers drives patients to other facilities), changes in the underlying population (shifts in population size or age), and changes in the local economy. Moving averages were not used because although they generate visually pleasing curves they smooth over day-of-week and seasonal effects that are important for developing baselines.

Concerning model quality, one useful test is whether the forecast error variance in the testing data is approximately the same as that in the training data. Upon dividing the forecast errors in the test data by their standard deviations in the training data, the scaled forecast error variances range from 0.87 to 1.07 for the seven CC categories (ideally, these ratios should be near 1). Further, the fraction of scaled forecast errors that exceed 1.96 ranged from 0.0 to 0.033 (when the model holds and residuals are Gaussian, the portion of one-sided residuals exceeding 1.96σ is 2.5%). Thus, departures from stationarity in the time series are mild enough that the forecast errors show that future complaints can be reasonably well predicted using a single baseline model for each category.

When monitoring complaint levels over multi-year time frames, it is necessary to periodically update baseline model coefficients in order to minimize the extrapolation in forecasting. One approach to choosing an update frequency is to do a planned update every year, but also monitor residuals for patterns, including shifting variance, that have not been observed previously to check whether additional updates are needed.

### Hierarchical modeling to capture season-to-season differences

The first-order model is useful for routine monitoring. It has the obvious shortcoming, however, of describing each season in a one-size-fits-all fashion. As noted above in evaluation of the model's goodness-of-fit, forecast errors reflect modelling imperfections as well as random variability, limiting somewhat the sensitivity of surveillance to detect smaller anomalies. Improving on this situation requires more refined baselining.

Hierarchical methods [23] can overcome the one-size-fits-all shortcoming, or, at a minimum, provide information that is valuable in assessing the quality of one-size-fits-all modelling assumptions. In the hierarchical approach, each season is allowed to have its own time of peak activity, its own seasonal duration, and its own peak magnitude. For practical purposes, the hierarchical model shares the global characteristics of the first order cyclical regression model. The seasonal component is modelled with a scalable Gaussian function, in contrast with the fixed-width sine and cosine harmonics previously. And the underlying baseline changes linearly within a season, as opposed to behaving linearly over a longer time period.

Applying the hierarchical model to respiratory CC data illustrates the season-specific nature of chief complaints. On the average, our respiratory complaints peak on January 22, with a season-to-season standard deviation in the day of the peak of 12 days. The durations of individual seasons, defined in terms of the standard deviations for the Gaussian-shaped peaks, vary by factor of two over the monitoring period. And there is no apparent relation between the time that the peak occurs and the magnitude of the flu season.

Use of hierarchical models for real time syndromic monitoring could be considered, but at a significant computational cost. In order to capture the peak time and magnitude of an ongoing season, the model must be updated on a frequent (e.g., weekly) basis, involving lengthy runs of Markov chain Monte Carlo software. Because the first order cyclical regression model fits the data sufficiently well to detect anomalies of interest, we have used the first order model for routine monitoring. A similar first order cyclical regression model is used by the Centers for Disease Control to monitor pneumonia and influenza related mortality data [24], also with success.

### Related efforts

Influenza surveillance basing alerts on comparison to historical data were described by Irvine [25]. Daily counts were compared to historical averages and standard deviations. Their data demonstrated a peak in CCs during influenza season.

Lazarus et. al. [26] use a generalized linear mixed model based on four years of data from ambulatory health encounters. They find that indicators for day-of-week, month, holiday effects as well as a secular trend term contribute significantly to their model fit. There may be ED data from other hospitals where month-to-month effects exist but are not part of a longer seasonal trend, but we don't see them in our data. Logistic regression [26] is useful for scaling over census tracts of different population sizes and, when complaint counts behave proportional to underlying census populations, is also useful in modeling overall complaint levels.

Reis and Mandl [27] used CCs for their time series models (autoregressive integrated moving average, ARIMA, models) for total and respiratory visits. After fitting a day-of-week effect and a seasonal effect, there remained positive autocorrelation in the forecast errors, which they modelled using a particular time series model. In our CC data, there is negligible autocorrelation in the errors after fitting our model, except that due to the variation in when the seasonal peak occurs. For example, if a peak occurs early, then we observe a sequence of positive errors, which leads to positive autocorrelation of the type reported. For our CC data, the best-fitting ARIMA-type model applied to the residuals after fitting the trend, seasonality, and day-of-week effect was the EWMA (equivalent to a moving average fit to the first differences, also denoted ARIMA(0,1,1) for the particular autoregressive integrated moving average model that it corresponds to). Reis and Mandl [27] note that the ARIMA model adjusts to multi-day outbreaks and so it reduces the error on days 2, 3, ... of a multi-day outbreak. Therefore they suggest using both the original errors (containing serial correlation) and the ARIMA-model-adjusted errors in two monitoring schemes.

The EWMA also adjusts to multi-day outbreaks and therefore suffers from signal loss if the outbreak persists for multiple days (the results in Table 2 illustrate this effect). Therefore, the Reis and Mandl [27] suggestion to monitor two residual series is relevant if we use EWMA or any approach (including the hierarchical model) that uses the very recent past (in addition to the trend, seasonality, and day-of-week effects) to modify the current forecast (leading to two or more forecast methods). Also, sequential tests were not used in [27] for their 7-day simulated outbreaks. Each simulated outbreak added simulated additional counts to the daily CC data. Forecasts were made (on the basis of a model that used the overall mean, the day-of-week means, and the trimmed day-of-year means) and were modified using the ARIMA-modeling of the residuals. If any single-day forecast error exceeded a threshold, then the simulated outbreak was said to be detected. Sequential tests are ideal for multi-day outbreaks so the performance (the false negative rate for a fixed false positive rate) of Page's statistic or a moving window such as in [28] or the scan statistic such as in [29] would be better than the performance of one-at-a-time tests in the case where all simulated outbreaks lasted 7 days. Reis et al. [28] applied several sliding detection windows, each of at most 7 days to ED visit daily counts in which simulated outbreaks (in the form of additional ED visits) lasting 3, 7, and 14 days were added to the real data in a simulation study. On the other hand, if each outbreak lasted only one day, then monitoring single-day errors would be optimal. In summary, we concur with [30] regarding the robustness and simplicity of Page's test. Alternatively, there are occasions when using a modest number of specific tests is effective as was done in [29].

### Use of free text chief complaints

Many surveillance systems report the use of CCs or discharge diagnoses based on ICD-9 codes. Most of these use discharge diagnosis ICD-9 codes in specialized settings such as the Military [13] or in HMOs [23]. ED ICD-9 codes were also used when processing data retrospectively [4]. In most EDs, however, ICD-9 discharge diagnosis coding is not performed on a "near-real" time basis and would not be available for "near-real time" surveillance.

By contrast, free text chief complaints are obtained at the time of patient entry into all Emergency Departments and free text discharge diagnoses are determined at or close to the time of ED discharge. Therefore, use of our grouping scheme is relevant to the majority of EDs in which chief complaints and discharge diagnoses are recorded as free text and are available for "near-real time" surveillance.

Because of their timeliness, CCs are used in our "near-real time" surveillance system B-SAFER [31,32]. We considered using CoCo, a naive Bayesian free-text classifier developed by the University of Pittsburgh [33], but this was not made available to us. Another automated classification based on weighted key words system is used by ESSENCE [34]. The New York City Department of Health uses a key word and key phrase SAS-based coding system [35]. A comparison of the performance of expert based classification systems such as ours, and automated classification systems has not been done.

There are other potential limitations in using ICD-9 codes for surveillance. It is to be expected that early cases of unusual diseases will be misdiagnosed. Assigned ICD-9 diagnostic codes may be more reflective of the diagnostic bias or practice patterns of the provider, than of the true incidence of disease. Furthermore, ICD-9 diagnosis code assignment is potentially subject to billing bias: codes which garner the highest reimbursement may be used, rather then those that most accurately represent the disease process. Use of ICD-9 codes for chief complaints is also problematic. Because ICD-9 codes were developed for classification of diagnoses, the dictionary for chief complaints is not robust. Therefore, the use of free text chief complaints may result in increased sensitivity, although the B-SAFER team believes that some types of coding standards would be beneficial [36].

### Results and applications

Day-of-week patterns in EDs have been previously reported in the literature [4,26,27,37,38]. Although the magnitudes of the day-of-week effects vary depending on the setting, the first day of the work week typically exhibits the greatest number of events. And, as we have shown, there is day-of-week variability within infectious disease syndromes which can be obscured by the failure to consider each body system's pattern individually. It is also important to understand utilization patterns on weekends, which ED data provides, as the effects of bio-terrorist or natural outbreaks are unlikely to be limited to weekdays.

Seasonal effects in infectious diseases are best known for respiratory infections (e.g. influenza and respiratory syncytial virus). This seasonality is in large part due to the yearly winter influenza epidemics. Our data is quite consistent with these findings. However, we provide an analysis of the pattern of respiratory related chief complaints based on the longest (8.6 years) historical data base. Furthermore, the seasonality of infectious disease complaints for other body systems, or for non-ID related complaints has not previously been reported. Seasonality in specific GI infections has been noted in other settings. Some gastrointestinal infections are more common in the winter (e.g. rotavirus) while others (e.g. *Campylobacter*, *Cryptosporidium*, enterovirus) are more common in the warm months from outdoor cooking and recreational water exposures.

Seasonality is also important because the signal-to-noise ratio in complaint counts maychange depending on time of year. As illustrated in Figure 1, the error bars are larger during the height of flu season than at other times of the year, leading to reduced sensitivity for detecting an increase in respiratory complaints at that time.

It is unclear why, in our data, the number of respiratory complaints fell with time while the number of fever complaints rose. This may reflect the relative mildness of recent influenza seasons. Alternatively, rather than actual differences in patient presentations, this may represent changes in the practices of choosing or recording chief complaints. This would require further investigation. Implementation of a standard drop-down menu for chief complaints might prevent some bias over time in the selection of chief complaints. Use of a new system, however, would likely change the distribution of chief complaints and thus not allow for the creation of baselines based on historic data.

### Surveillance

Using the models described above we successfully identified a respiratory outbreak in advance of the traditional flu-reporting data streams described in the Methods section. Incoming B-SAFER reports were monitored at least once daily, seven days a week, by the project epidemiologist. This allowed for prompt handling of events indicating a condition reportable by statute to the NM Department of Health. Because there is approximately a 2-week delay for traditional flu-related data sources, provided our respiratory CC captures some of the NM flu cases, we expected to, and did, identify a flu-related respiratory peak in advance of these other sources.

### Limitations

This analysis is based on data from one ED and patterns identified may be somewhat specific to metropolitan Albuuerque. Indigent or Hispanic populations may be over-represented in the ED studied as compared to other EDs. Visit patterns may differ by the local health care infrastructure, population insurance status, access to care, or local climate. We wewere fortunate that electronic ED data was available for the previous eight years. Other institutions may lack the source data for a similar analysis.

CCs are determined by a nurse and recorded in free text by a clerk. This process may conceivably distort patients' literal CCs. Free text CCs are quite variable and require extensive processing. These CCs may also have varied had they been recorded by a physician. Although the rationale for using CCs rather than discharge diagnoses was provided above, there is a tradeoff between the better timeliness of CC data and the better sensitivity of discharge diagnoses [39]. Note that our modelling is as easily applied to diagnoses codes as to chief complaints.

Any approach to disease surveillance using either CCs or discharge diagnoses requires large numbers of symptomatic patients. Analyses based on such large-scale counts are unlikely to discover a small and geographically dispersed event such as the anthrax Anthrax outbreak of October 2001.

As we work more with our data, we will understand it better. Opportunities exist for performing sensitivity analyses, comparing the baseline patterns for CCs to those for discharge diagnoses, and more thoroughly evaluating the performance of our signals as compared to existing standards.

## Conclusion

We have demonstrated a robust statistical approach to characterize baseline data for ED visits. We demonstrated day-of-week, seasonal and long-term effects by infectious disease in grouped chief complaint categories. ED data provides information on daily visit patterns, rather than just 5-day-a-week patterns. Using respiratory complaints as an example, we have shown that these models when applied to "near real-time" surveillance data provide an early indicator of an anomaly. This increase in respiratory visits was identified early by a rise in Page's statistic. This anomaly corresponded to events detected later by more traditional methods. Understanding baseline patterns in ED data provides the ability to distinguish expected versus unexpected events during infectious disease surveillance.

## Competing interests

The author(s) declare that they have no competing interests.

## Authors' contributions

JB and EU provided medical advice from the ED and public health perspectives. They were responsible for choice and groupings of chief complaints. JB obtained IRB approvals and recruited sites. TB and RP are responsible for choice and implementation of statistical tests. DF provided computer systems, interface and architecture. EJ provided technical input, review and project direction. JB, EU, TB and RP collaboratively wrote the manuscript. All authors read and approved the final manuscript.

## Pre-publication history

The pre-publication history for this paper can be accessed here:


